# Evidence supporting endodontic therapy as a safe strategy in patients previously treated with head and neck radiotherapy: a scoping review

**DOI:** 10.1007/s00520-026-11139-2

**Published:** 2026-08-31

**Authors:** Álvaro Souza Wanderley, Wesley Viana de Sousa, Maria Olívia da Costa, Alícia Marcelly Souza de Mendonça Silva, Raylane Farias de Albuquerque, Igor Henrique Morais Silva, Lucas Nascimento Ribeiro, Diana Santana Albuquerque, Marlos Barbosa-Ribeiro

**Affiliations:** 1 School of Dentistry of Pernambuco, Postgraduate Program in Dentistry, University of Pernambuco, Recife, PE 50040-200 Brazil; 2 Department of Dentistry at the Pernambuco Cancer Hospital, Recife, PE 50040-000 Brazil; 3 School of Dentistry of Arcoverde, University of Pernambuco, Campus Arcoverde, Rua Cícero Monteiro de Melo, S/N, São Cristóvão, Arcoverde, PE 56503-146 Brazil

**Keywords:** Endodontics, Radiotherapy, Head and neck cancer, Osteoradionecrosis

## Abstract

**Purpose:**

To map and synthesize the available scientific evidence supporting the effectiveness and safety of endodontic therapy in patients who underwent head and neck radiotherapy, considering clinical and radiographic outcomes and treatment-related complications, particularly osteoradionecrosis (ORN).

**Methods:**

This scoping review followed the Arksey and O’Malley framework, informed by the Joanna Briggs Institute Manual for Evidence Synthesis, and was reported according to PRISMA-ScR guidelines. Comprehensive searches were conducted in PubMed, Embase, Scopus, Web of Science, MEDLINE, LILACS, and gray literature sources, with no date restrictions. Clinical studies evaluating endodontic therapy in irradiated patients were included. Data were charted and narratively synthesized using a Population–Concept–Context framework. Consistent with scoping review methodology, no critical appraisal was performed.

**Results:**

Of 174 identified records, five studies met the inclusion criteria. Reported success rates ranged from 18 to 100%, with lower rates observed in earlier studies employing outdated techniques. More recent investigations demonstrated high clinical success, improved radiographic stability, and no reported cases of ORN. Across all studies, endodontic therapy was not associated with ORN. However, methodological heterogeneity was noted, particularly regarding outcome definitions and follow-up duration.

**Conclusion:**

Endodontic therapy appears to be a safe and conservative treatment option for patients with head and neck cancer who underwent radiotherapy and may reduce the need for invasive procedures associated with ORN risk. However, the limited and heterogeneous evidence highlights the need for well-designed prospective studies to support standardized clinical decision-making within supportive oncology care.

## Introduction

Head and neck cancer represents a major global health burden, characterized by substantial incidence rates, significant mortality, and persistent challenges related to early diagnosis [1]. According to the International Agency for Research on Cancer of the World Health Organization, malignancies arising in the head and neck region rank among the most frequently diagnosed cancers worldwide [2]. In 2022, GLOBOCAN estimated a global incidence of head and neck cancer of approximately 940,000 new cases and about 480,000 deaths, corresponding to nearly half the number of newly diagnosed cases [3]. In Brazil, projections from the National Cancer Institute estimate approximately 781,000 new cases in 2026 [4]. The multifactorial etiology of head and neck cancer, combined with the absence of reliable early diagnostic markers, contributes to the high proportion of cases diagnosed at advanced stages. Consequently, nearly 80% of patients present with late-stage disease, a scenario that dramatically compromises prognosis, with 5-year survival rates declining from approximately 95% in early-stage tumors to nearly 30% in advanced presentations [5, 6].

Following the diagnosis of head and neck cancer, patients are informed about available therapeutic options, including surgery, chemotherapy, radiotherapy, or combined treatment strategies [7]. Radiotherapy remains one of the most widely employed modalities in contemporary oncologic care and is commonly associated with systemic and oral adverse effects [8]. Head and neck radiotherapy can cause several oral complications, including mucositis, trismus, xerostomia, radiation-associated tooth decay, and osteoradionecrosis (ORN), which can significantly compromise patients’ oral function and quality of life [9, 10]. Radiation-induced caries primarily results from salivary hypofunction, causing alterations in the oral microbiota and impairing biofilm control, which may progress to extensive infectious processes and ultimately require invasive dental interventions, including tooth extraction or endodontic therapy [11].

Currently, tooth extraction prior to radiation therapy is often recommended as a strategy to control odontogenic infections associated with teeth with pulpal and/or periapical disease in these patients. Some narrative studies on mandibular ORN have reinforced that ORN is still a recalcitrant skeletal complication of orofacial cancer radiotherapy that requires adequate clinical planning before therapy [12–14]. Contemporary clinical guidelines, including those issued by the ISOO–MASCC–ASCO, advocate for dental extractions prior to the initiation of radiotherapy in an effort to minimize future complications [15, 16]. Although ORN is a serious radiation-related complication and can be triggered by trauma or surgical procedures on previously irradiated tissues, it is unlikely to develop after an extraction performed before radiotherapy, provided that adequate healing time is ensured before the start of radiotherapy [15, 17].

In non-oncologic patients, endodontic therapy is widely regarded as the treatment of choice for managing oral infectious foci whenever feasible, as it allows preservation of the affected tooth, thereby preventing tooth loss and the subsequent need for prosthetic rehabilitation [16, 18]. Emerging evidence suggests that endodontic therapy may also represent a viable alternative for eliminating odontogenic infectious foci associated with pulpal and/or periapical disease in patients who underwent head and neck radiotherapy [19]. Recent studies have reported favorable clinical outcomes, characterized by high success rates and the absence of ORN, positioning endodontic therapy as a potentially superior approach to tooth extraction, which may be associated with long-term morbidity, the need for complex rehabilitation, and, in certain circumstances, delays in oncologic treatment with potentially life-threatening consequences [20].

Despite advances in oncologic therapies and dental management for head and neck cancer [21, 22], the evidence supporting endodontic therapy in irradiated patients remains fragmented and heterogeneous, particularly with respect to clinical and radiographic outcomes and treatment safety, including the risk of ORN. This uncertainty continues to influence clinical decision-making and often favors more invasive approaches. Therefore, this scoping review aimed to map, characterize, and synthesize the available scientific evidence on the effectiveness and safety of endodontic therapy as a conservative approach for managing odontogenic infectious foci associated with pulpal and/or periapical disease in patients with head and neck cancer who underwent radiotherapy.

## Materials and methods

This scoping review was conducted in accordance with the five-stage methodological framework [23], comprising the following: identification of the research question; identification of relevant studies; study selection; data charting; and collation, summarization, and reporting of results. The guiding research question was as follows: “What evidence is available regarding the effectiveness and clinical and radiographic outcomes of endodontic therapy in patients who underwent head and neck radiotherapy?”

The methodological approach was further informed by the Joanna Briggs Institute Manual for Evidence Synthesis [24] and reported in accordance with the Preferred Reporting Items for Systematic Reviews and Meta-Analyses *Extension for Scoping Reviews* (PRISMA-ScR) [25]. The review protocol was prospectively registered in the Open Science Framework under the registration https://doi.org/10.17605/OSF.IO/T5YNV.

### Eligibility criteria

Eligibility criteria were based on the search question, and the PCC strategy used to guide the selection of search terms was as follows: population (patients who underwent radiotherapy to the head and neck region), concept (endodontic therapy), context (assessment of treatment outcomes).

This review included articles involving adult patients diagnosed with head and neck cancer, of any subtype, in whom radiotherapy was the treatment of choice for the disease and who subsequently underwent endodontic therapy. There were no restrictions regarding year of publication, and observational studies, retrospective studies, case reports, and case series were included. Exclusion criteria included in vitro or animal studies, as well as non-original or non-peer-reviewed publications. Studies in which endodontic therapy was performed prior to radiotherapy were also excluded, as the aim of this review was to evaluate the clinical success of endodontic therapy radiotherapy to the head and neck region, as well as the risk of ORN associated with this procedure.

### Information sources and search strategy

The following bibliographic databases were used: Medline/PubMed, Embase, Scopus, Web of Science, and LILACS. The gray literature was included using Google Scholar and ProQuest. The search strategy was elaborated on the PubMed platform and adapted to other databases, using the terms: (“Osteoradionecrosis”[MeSH Terms] OR osteoradionecrosis* OR ORN) AND (“Radiotherapy”[MeSH Terms] OR radiotherap* OR irradiation OR radiotherapy-induced) AND (“Endodontics”[MeSH Terms] OR endodontic* OR “Root Canal Therapy”[MeSH Terms] OR root canal*) AND (“Head and Neck Neoplasms”[MeSH Terms] OR “head and neck cancer*” OR “oral cancer*” OR “oropharyngeal neoplasm*”). MeSH terms were also included to broaden the literature search. No limitations regarding language, year, or type of publication were applied to the search. The strategies used for each database are listed in Table 1.

**Table 1:** Search strategy

Database	Search strategy	Filter
PubMed	(“Osteoradionecrosis”[MeSH Terms] OR osteoradionecrosis* OR ORN) AND (“Radiotherapy”[MeSH Terms] OR radiotherap* OR irradiation OR radiotherapy-induced) AND (“Endodontics”[MeSH Terms] OR endodontic* OR “Root Canal Therapy”[MeSH Terms] OR root canal*) AND (“Head and Neck Neoplasms”[MeSH Terms] OR “head and neck cancer*” OR “oral cancer*” OR “oropharyngeal neoplasm*”)	No filters applied
Scopus	TITLE-ABS-KEY(osteoradionecrosis*) AND TITLE-ABS-KEY(radiotherap* OR irradiation) AND TITLE-ABS-KEY(endodontic* OR “root canal” OR “root canal therapy”) AND TITLE-ABS-KEY(“head and neck cancer*” OR “oral cancer*” OR “oropharyngeal neoplasm*”)	No filters applied
Web of Science	TS=(osteoradionecrosis*) AND TS=(radiotherap* OR irradiation) AND TS=(endodontic* OR “root canal” OR “root canal therapy”) AND TS=(“head and neck cancer*” OR “oral cancer*” OR “oropharyngeal neoplasm*”)	No filters applied
LILACS	(osteoradionecrosis OR osteorradionecrose) AND (radiotherapy OR radioterapia) AND (endodontics OR endodontia OR “root canal”) AND (“head and neck cancer” OR “neoplasias de cabeça e pescoço” OR “câncer de boca” OR “oral cancer”)	No filters applied
Embase	(“osteoradionecrosis”/exp OR osteoradionecrosis* OR ORN) AND (“radiotherapy”/exp OR radiotherap* OR irradiation OR “radiation therapy”) AND (“endodontics”/exp OR endodontic* OR “root canal therapy”/exp OR “root canal”) AND (“head and neck neoplasm”/exp OR “head and neck cancer” OR “oral cancer” OR “oropharyngeal neoplasm”)	No filters applied
Google Scholar (gray literature)	(“head and neck” OR “cabeça e pescoço”) AND (“radiation therapy” OR radioterapia) AND (“endodontic treatment” OR “tratamento endodôntico” OR “root canal therapy”) AND (“success rate” OR “treatment outcome” OR “sucesso clínico” OR “sucesso radiográfico”) AND (“thesis” OR “dissertation” OR “teses” OR “dissertação” OR “monografia”)	No filters applied

Two authors working independently (ASW and WVDS) reviewed the abstract and title of the search results. After searching each database, duplicate studies were removed using Rayyan software [26]. All potentially relevant articles were investigated by reading the full text. If there was a difference of opinion, consensus was reached by consulting a third author (MBR). To assess the agreement between the evaluators for the inclusion of the studies, the kappa coefficient was used [27]. Data extraction was also carried out by the two independent evaluators (ASW and WVDS); differences in results were discussed and resolved by a third evaluator (MBR).

The variables were analyzed using a standardized form including author and year, study design, sample size, radiation dose, timing of radiotherapy, endodontic preparation, irrigant type, obturation technique, sealer type, outcomes, and results.

### Outcome assessment

The objective of the review was to identify and characterize the interventions described in the literature regarding endodontic therapy in patients who underwent radiotherapy to the head and neck region. Studies describing endodontic protocols, such as the type of instrumentation used, irrigating solutions, and obturation techniques, were included. In addition, studies reporting radiation dose and the time elapsed after ionizing radiation therapy were considered. Studies that reported clinical outcomes (normalization of the periodontal ligament, periapical stability, and retention of the tooth, as well as the occurrence of ORN), as well as those describing the impact of these interventions on patients’ quality of life and overall well-being, were also included.

### Critical appraisal of individual sources of evidence

To characterize the overall strength of the evidence mapped in this scoping review, each included study was classified according to its methodological design using a standardized evidence hierarchy. The evidence quality classification proposed by Stetler et al. [28], based on an adaptation of the Agency for Healthcare Research and Quality (AHRQ) system, was employed. Two reviewers (ASW and MOC) independently classified each included study. The results of this classification were presented narratively and visually to illustrate the distribution of evidence levels in the literature on endodontic interventions in patients who underwent radiotherapy. This analysis was not used to exclude studies but rather to inform the discussion and provide context regarding the robustness of the available evidence.

## Results

The electronic search yielded a total of 174 records across the selected databases and sources. Following the removal of duplicate records using the Rayyan QCRI platform and a rigorous screening process, five studies met the eligibility criteria and were included in this scoping review [29–33]. The included evidence comprised three case series [29, 30, 33], one retrospective cohort study [31], and one case report [32]. The study selection process demonstrated a high level of inter-reviewer reliability. Cohen’s kappa coefficients indicated near-perfect agreement between reviewers across databases, with values of 0.86 for PubMed/MEDLINE, 0.80 for Scopus, and 0.87 for the remaining databases.

**Fig. 1:**
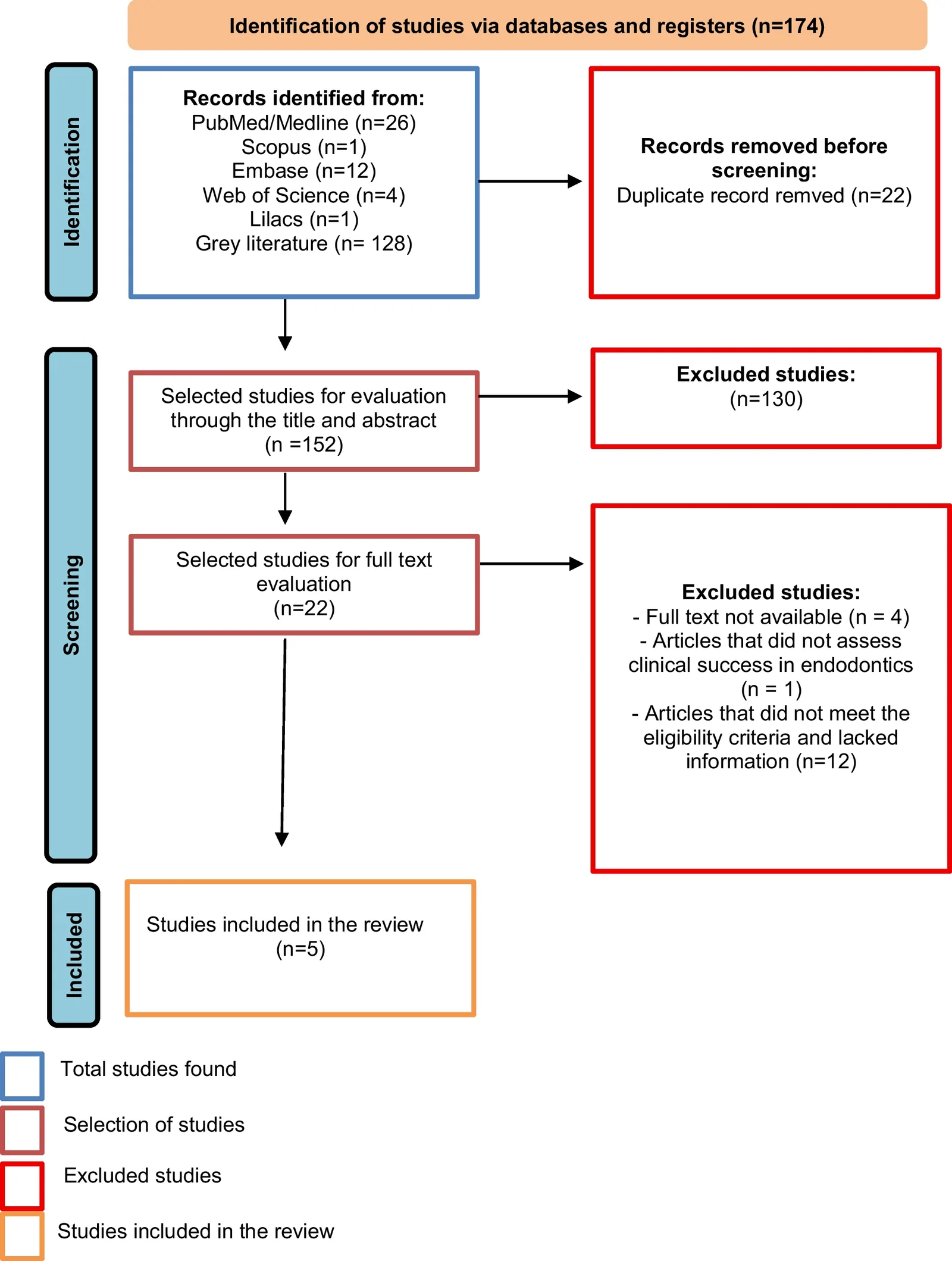
Flowchart of the search in the database

To facilitate transparency and rapid understanding of the evidence, a PRISMA flowchart summarizing the study selection process was constructed (Fig. 1), and a descriptive table was developed. Table 2 presents a comprehensive synthesis of the included studies, detailing authorship, year of publication, sample characteristics, primary diagnosis and tumor location, radiotherapy technique, radiation dose, timing of radiotherapy, endodontic preparation protocols, irrigation strategies, obturation techniques and obturation cements, and reported clinical outcomes and complications.

**Table 2:** Summary of included studies evaluating the success rate of endodontic treatment and the presence of osteoradionecrosis

Authors (year) (country)	Study design	Sample	Diagnosis/location	Radiotherapy technique	Radiation dose	Timing of radiotherapy	Endodontic technique	Irrigating substance	Obturation technique	Sealer	Outcomes and results
Montgomery [32] (USA)	Case report	1 patient (multiple teeth)	Undifferentiated squamous cell carcinoma of the nasopharynx with cervical metastasis	Cobalt 60	5950 rads in the oropharynx; 5904 rads in the nasopharynx; 5490 rads in the cervical region	Years after RT	Manual mechanical preparation	NR	NR	NR	Infection control, no ORN, extractions avoided
Markitziu and Heling [29] (Israel)	Case series	2 patients/11 teeth	Case 1, nasopharyngeal carcinoma; Case 2, histiocytic lymphoma in the left tonsil	Case 1: NR; Case 2, cobalt	Case 1, 11,000 rads; Case 2, 7000 rads	Post-RT (2 years and 6 months)	Manual mechanical preparation	Saline solution	Lateral condensation	AH-26	Sucess 2/11 (~ 18%), no ORN, antibiotics prescribed for 5 days
Seto et al. [30] (USA)	Case series	16 patients/35 teeth (54 roots)	NR	NR	10 patients, > 6500 rads; 6 patients, 4500 rads and 6500 rads	Post-RT (6–54 months)	NR	NR	Short vs normal/long obturation (length analysis)	NR	85% success (46/54), 0 ORN; short obturation, fewer periapical changes
Lilly et al. [31] (USA)	Retrospective cohort	11 patients/22 teeth	10 patients, squamous cell carcinoma; 01 patient, rhabdomyosarcoma	NR	≥ 5000 cGy	≥ 6 months post-RT, follow-up 19 months	Step-back technique	NR	Lateral condensation	Sealer Grossman	91% success, 0 ORN, normalization PDL
Castagnola et al. [33] (Italy)	Contemporary case series	8 patients/10 teeth	3 patients, squamous cell carcinoma (SCC) of the oropharynx; 1 patient, oral SCC; 2 patients, laryngeal SCC; 1 patient, olfactory neuroblastoma	NR	Mean total dose, 66.25 Gy; average periapical dose, 39.36 Gy	137 days after RT (1–282 days)	Rotary ProTaper Next	NaOCl 5,25%, EDTA 17%, saline	Single-cone technique	AH Plus	100% success, 0 ORN, trismus limited clinical access

*NR* not reported

**Fig. 2:**
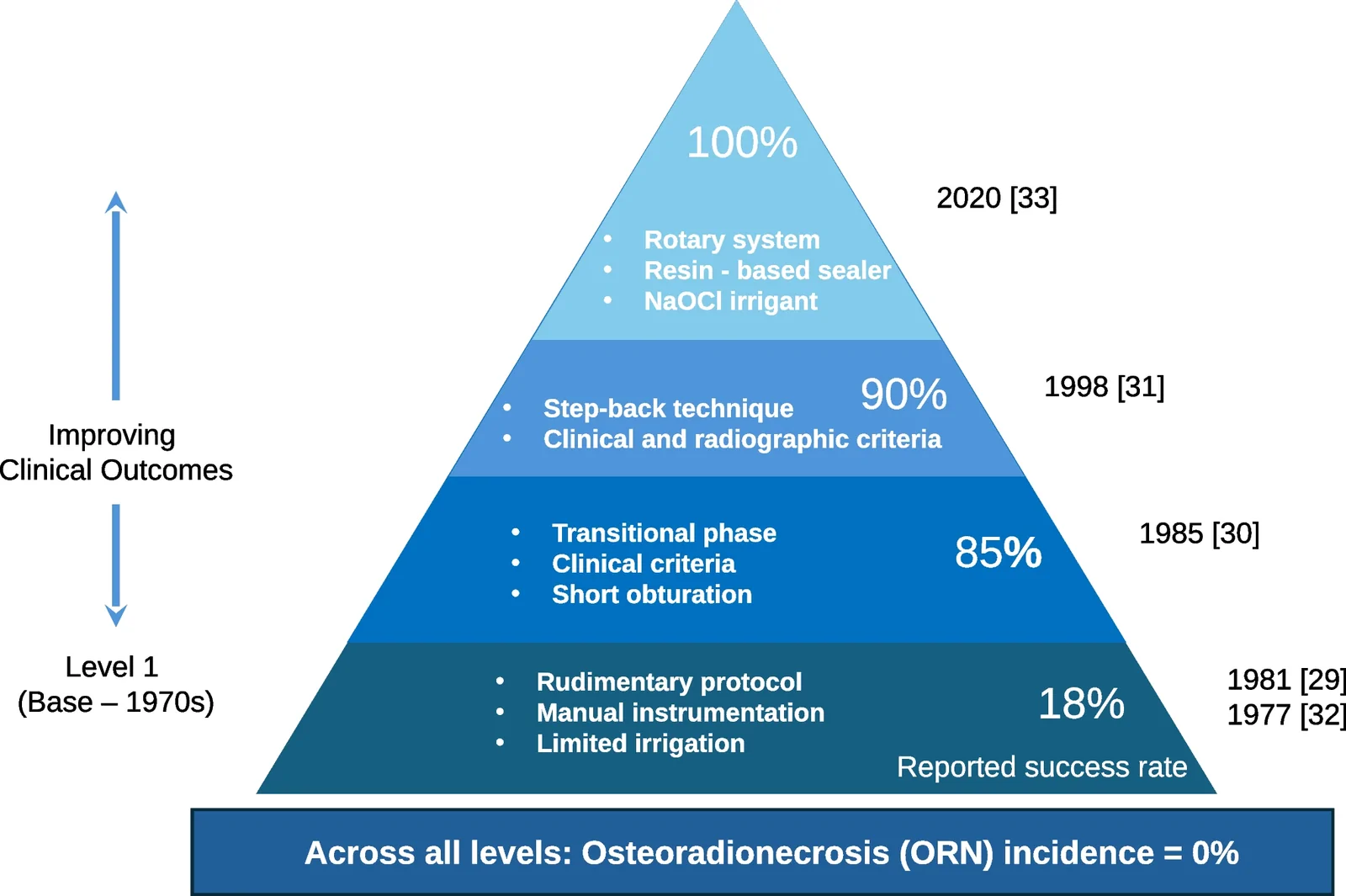
Temporal evolution of endodontic therapy protocols and reported clinical outcomes in patients undergoing head and neck radiotherapy, illustrating the association between protocol optimization and increasing success rates

The included studies predominantly involved patients diagnosed with squamous cell carcinoma affecting the nasopharynx, oropharynx, oral cavity, and larynx. When reported, conventional external beam radiotherapy with Cobalt-60 was employed in older studies, while more recent investigations provided limited technical details but reflected contemporary radiotherapy dose protocols. In general, the data demonstrate that endodontic therapy was performed on teeth located in regions previously exposed to radiotherapy for head and neck tumors, frequently involving anatomical sites considered high-risk for the development of ORN. A synthesis of these clinical findings, including the evolution of endodontic therapy in irradiated patients, the occurrence of ORN, and overall success rates, is illustrated in Fig. 2.

### Clinical and radiographic outcome assessment

Outcome assessment varied among the included studies. Earlier investigations relied predominantly on clinical parameters, such as infection control, symptom resolution, and tooth preservation, due to limitations in radiographic interpretation of irradiated bone [29, 30, 32]. In contrast, more recent studies incorporated combined clinical and radiographic evaluation, including assessment of periodontal ligament normalization and periapical stability [31, 33]. This progression reflects an evolution toward more comprehensive outcome assessment methods in contemporary endodontic practice.

### Radiation parameters and treatment timing

**Fig. 3:**
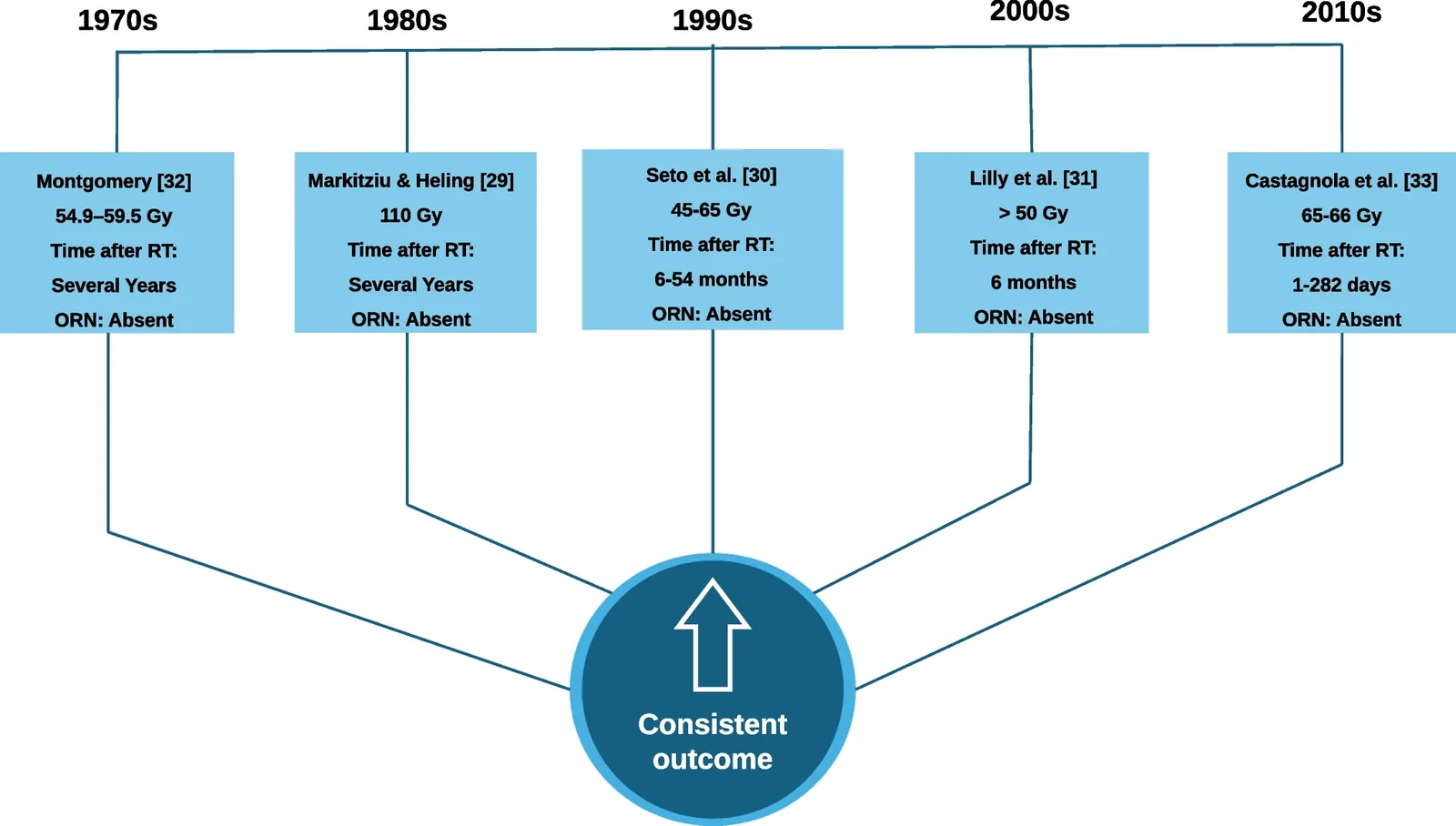
Timeline of endodontic treatment outcomes in patients undergoing head and neck radiotherapy across different decades. The figure summarizes key studies from the 1970 s to the 2010 s, highlighting radiation doses, time elapsed after radiotherapy, and the occurrence of osteoradionecrosis (ORN)

Radiation doses ranged from approximately 4500 rads to more than 11,000 rads in earlier studies [29, 30, 32], whereas later investigations reported total doses expressed in gray, including values ≥ 5000 cGy [31] and contemporary protocols of 65–66 Gy with a mean periapical exposure of 39.36 Gy [33]. Endodontic therapy was performed at variable intervals following radiotherapy, ranging from several years after completion [29, 32] to a few months post-radiotherapy [30, 31] and as early as 1 day after completing the treatment [33]. Despite these variations in radiation dose (reported in rads, cGy, or gray) and timing of intervention, none of the included studies reported ORN associated with endodontic therapy, suggesting consistent clinical stability over time (Fig. 3).

### Endodontic techniques and clinical outcomes

Endodontic protocols differed substantially across studies. Historically published investigations employed manual instrumentation, limited irrigation strategies, and conventional obturation techniques, resulting in lower or inconsistent success rates (18%) [29, 32]. Seto et al. emphasized apical control during obturation and reported improved outcomes (~ 85%) [30]. With further standardization of endodontic procedures, including step-back preparation, lateral condensation, and the use of appropriate sealers, Lilly et al. [31] reported success rates exceeding 90%. The most recent study, conducted by Castagnola et al. [33], using rotary instrumentation, sodium hypochlorite and EDTA irrigation, and single-cone obturation with epoxy resin–based sealer, reported complete clinical success without cases of ORN.

### Critical analysis of the evidence

**Fig. 4:**
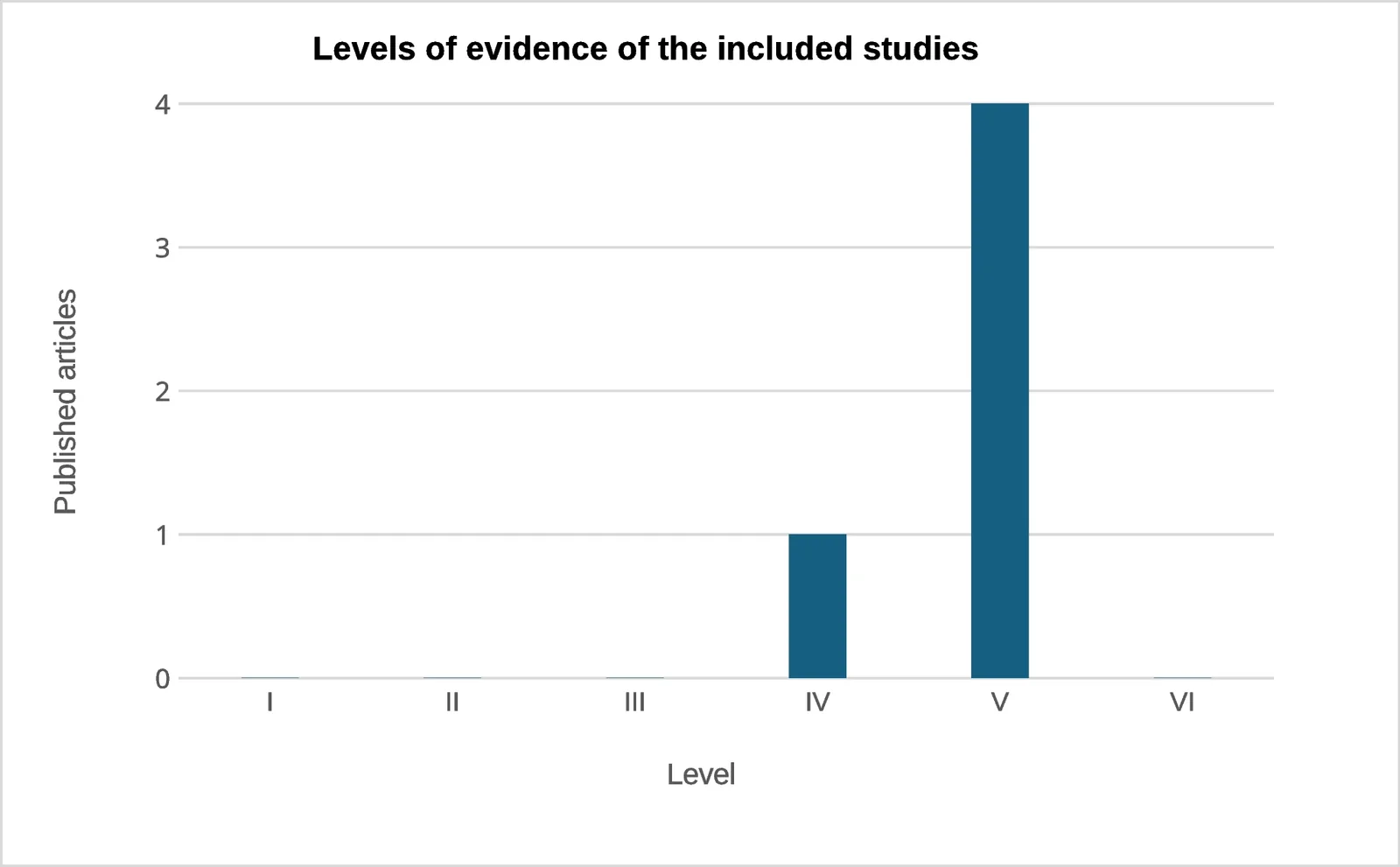
Classification of the included studies according to levels of evidence based on the system proposed by Stetler et al. (1998)

The studies included in this scoping review predominantly presented a low level of evidence according to the hierarchy proposed by Stetler et al. [28]. There was a predominance of nonexperimental observational designs, including one case report [32] and three case series [29, 30, 33], which correspond to level V evidence. These designs are characterized by descriptive methodology, the absence of control groups, and limited sample sizes. Only one study [31] employed a retrospective cohort design and was therefore classified as level IV evidence. No controlled clinical trials or quasi-experimental studies were identified. This highlights a substantial gap in higher-level evidence regarding the success of endodontic therapy and the risk of ORN in patients who underwent head and neck radiotherapy (Fig. 4).

## Discussion

This scoping review synthesized the available evidence on endodontic therapy as a safe and effective treatment option to avoid the extraction of teeth with infectious foci in patients who had previously undergone head and neck radiotherapy. Although the initial search retrieved a substantial number of records, only five studies met the eligibility criteria and were included in the qualitative synthesis [29, 33]. Despite the methodological limitations inherent to the available evidence, the findings suggest that endodontic therapy may represent a viable conservative alternative in previously irradiated patients. Currently, there is insufficient scientific support to routinely recommend tooth extractions based solely on a history of radiotherapy.

The frequency of dental extractions reported during the follow-up of patients who underwent radiotherapy in the head and neck region, associated with the wide variation in the interval between the end of radiotherapy and tooth removal, highlights the cumulative impact of radiation-induced oral complications, as well as limitations in access to specialized dental care [34]. A significant portion of these extractions is related to residual roots, advanced periodontal disease, and extensive radiation-associated caries lesions with pulpal involvement, frequently in patients who did not undergo endodontic therapy due to financial constraints or the unavailability of this procedure in public health services [35, 36]. This scenario reveals a significant mismatch between recommendations based on scientific evidence and daily clinical practice. Dental extractions in previously irradiated bone are recognized as one of the main risk factors for the development of ORN, since surgical trauma can compromise an already hypovascularized area with reduced tissue repair capacity, favoring the occurrence of this complication [37, 38].

In this context, prioritizing endodontic therapy is not only a conservative alternative but also a preventive strategy for potentially serious late complications. By promoting the elimination of odontogenic infection associated with pulpal and/or periapical disease, preserving the structure and function of the affected tooth, controlling painful symptoms, and preserving adjacent soft tissues and bone structures, endodontic therapy may reduce the need for surgical interventions in hypovascularized irradiated bone. Furthermore, it contributes to the maintenance of masticatory function and long-term oral stability in a population already vulnerable to hyposalivation, trismus, and accelerated progression of radiation-related caries [35, 36, 39]. Therefore, expanding access to endodontic therapy, especially in hospitals and the public oncology care network, may reduce the need for tooth extractions and, consequently, mitigate the risk of ORN, aligning clinical practice with contemporary guidelines for supportive oncology care [9, 22, 40].

Early clinical evidence provides important historical context for this therapeutic approach. The case report by Montgomery [32] represents one of the earliest demonstrations of successful endodontic therapy in irradiated tissues. At that time, endodontic therapy was performed under considerably less rigorous protocols, particularly regarding aseptic control, instrumentation, and obturation accuracy. Nevertheless, favorable clinical outcomes were achieved even in the presence of severe radiation-induced oral alterations.

Success criteria varied considerably across the studies. Earlier studies presented definitions that were less standardized or more lenient, regarding periapical stability, the absence of symptoms, or tooth retention as favorable indicators [29, 32]. In contrast, more recent studies applied stricter clinical and radiographic criteria, similar to those used for non-irradiated teeth, including the absence of signs or symptoms, the absence of a sinus tract, the maintenance of the periodontal ligament space, and the absence of periapical lesions [30, 31, 33]. This methodological heterogeneity makes it difficult to directly compare success rates across studies, although none reported ORN associated with endodontic therapy.

The absence of major complications, especially ORN, reported in this and subsequent studies supports the premise that endodontic therapy, when performed with appropriate technical adaptations and strict aseptic control, can be safely conducted in irradiated patients [29, 33]. These findings challenge the routine contraindication of endodontic therapy in irradiated patients and suggest that conservative approaches may represent a safe and clinically feasible option for managing teeth with pulpal and/or periapical disease in this population. Low success rates (approximately 18%) were reported [29], which were attributed to limitations such as imprecise manual instrumentation, saline-only irrigation, and the absence of standardized antibacterial protocols. Despite these constraints, no cases of ORN were observed, suggesting that endodontic therapy itself does not represent a direct risk factor for this complication.

Subsequent studies demonstrated progressively improved outcomes. Seto et al. [30] reported a success rate of approximately 85%, based primarily on clinical criteria, emphasizing the potential role of apical control during obturation in minimizing periapical alterations. With further standardization of endodontic techniques, Lilly et al. [31] reported success rates exceeding 90%, supported by combined clinical and radiographic evaluation. The most recent study included in this review, conducted by Castagnola et al. [33], utilized rotary instrumentation, sodium hypochlorite and EDTA irrigation, and single-cone obturation with an epoxy resin–based sealer, achieving complete clinical success without cases of ORN. Collectively, these findings suggest that modern endodontic protocols may be safely and effectively performed in patients previously treated with head and neck radiotherapy.

Overall, the available evidence indicates that endodontic therapy is both feasible and effective in irradiated patients, particularly when contemporary techniques and standardized protocols are employed [41]. Nevertheless, relevant methodological heterogeneity persists, characterized by a limited number of studies and a wide time span that reveals significant disparities in radiotherapy dosages and endodontic protocols, especially regarding outcome definitions and post-radiotherapy follow-up periods. Only two studies incorporated radiographic assessment [31, 33], reflecting the progressive evolution of methodological rigor in this field. Additional challenges remain regarding the impact of radiotherapy on dentin structure and its implications for endodontic procedures, including sealer adhesion [42]. Accurate diagnosis and minimally invasive approaches remain essential to ensure long-term success and preserve quality of life in patients treated with head and neck radiotherapy.

In this regard, when the endodontic protocol is strictly confined to the apical cemento-dentin-cementum junction, the likelihood of exacerbating periapical infections is drastically minimized, reinforcing the need for technical precision. Cone-beam computed tomography has emerged as a valuable adjunct for treatment planning, enabling three-dimensional assessment of root anatomy and improved detection of periapical alterations [43]. Given the structural changes induced by radiation in dental tissues, endodontic therapy in irradiated patients should be performed in an atraumatic manner to minimize inflammatory responses and optimize prognosis [29, 30]. Although ORN possesses a complex and multifactorial pathophysiology, strongly influenced by factors such as smoking and the oral microbiota profile, the progressive improvement in success rates over time, coupled with the absence of a direct association with ORN, suggests that endodontic therapy may serve as a safe, conservative approach within contemporary supportive cancer care, in accordance with current clinical guidelines [9].

Future studies should also consider anatomical and tooth-related factors that may influence both the risk of ORN and the outcomes of endodontic therapy. The mandible, particularly its posterior region, appears to be more susceptible to ORN than the maxilla; however, current guidelines emphasize that risk assessment should consider the radiation dose and volume received by the specific jaw region rather than anatomical location alone [15]. Therefore, future studies should stratify endodontic outcomes according to tooth location and, whenever possible, incorporate the radiation dose received by the corresponding jaw region. In addition, treatment success should be evaluated according to tooth type and anatomical complexity, particularly distinguishing complex multirooted molars from single- and two-rooted teeth. Such stratification could help clarify whether anatomical location, radiation exposure, and tooth complexity influence the safety and effectiveness of endodontic therapy in previously irradiated patients.

Accurate diagnosis is particularly important before initiating endodontic therapy in previously irradiated patients, as osteoradionecrosis may present with clinical and radiographic findings that overlap with those of odontogenic and periapical diseases, including bone destruction and radiolucent changes [44]. Therefore, radiographic abnormalities should not be interpreted in isolation but rather in conjunction with pulpal and periapical diagnostic tests, clinical findings, and the patient’s history of radiotherapy [44]. When conventional imaging does not provide sufficient information for diagnosis or treatment planning, three-dimensional imaging, such as cone-beam computed tomography (CBCT), may provide additional information regarding the extent and characteristics of periapical and osseous changes [45, 46]. This distinction is clinically relevant because misdiagnosing osteoradionecrosis as a primary endodontic lesion may lead to unnecessary interventions in previously irradiated tissues.

Although direct evidence linking rubber dam clamp trauma to ORN is lacking, caution during clamp placement may be warranted in previously irradiated patients. Gallego et al. reported a case in which trauma associated with a rubber dam clamp during endodontic treatment was considered a potential contributing factor to bisphosphonate-related osteonecrosis of the jaw, suggesting that localized mechanical trauma may represent a potential risk in patients with impaired bone healing [47]. More recently, Gozon et al. described cortical bone erosion and sequestration following the use of a stainless-steel rubber dam clamp during endodontic treatment in an otherwise healthy patient [48]. Although these reports do not directly establish a relationship between rubber dam clamp trauma and ORN, they support the rationale for careful clamp selection and placement in irradiated patients. This consideration may be particularly relevant in irradiated tissues, in which vascular, cellular, and tissue-healing alterations may compromise the response to local trauma. Therefore, the rubber dam clamp should be appropriately selected and positioned atraumatically, with particular attention to avoiding excessive compression or injury to the gingival and periodontal tissues.

The predominance of low-level evidence identified in this scoping review highlights the fragility of the current scientific foundation regarding endodontic therapy outcomes in irradiated patients. Most available data derive from case reports and small case series, which, although clinically valuable and hypothesis-generating, are inherently limited by small sample sizes, the absence of control groups, and restricted external validity. Only one retrospective cohort study [31] was identified, underscoring the scarcity of analytical designs capable of establishing stronger associations between radiotherapy exposure, endodontic management strategies, and the risk of ORN. Moreover, heterogeneity in radiotherapy parameters, particularly regarding dosage, as well as variations in endodontic protocols, outcome definitions, and follow-up periods further limits comparability across studies. Despite these limitations, the consistent absence of ORN associated with ET across the included reports provides clinically reassuring evidence. Nevertheless, well-designed retrospective and prospective cohort studies, along with multicenter investigations with standardized protocols, are urgently needed to strengthen the evidence base and support more definitive clinical recommendations.

## Conclusion

The available evidence suggests that endodontic therapy, when performed using contemporary techniques and materials, is consistently associated with favorable clinical outcomes and an absence of reported ORN in the included studies. However, the limited number of studies, the predominance of low-level evidence, and methodological heterogeneity highlight the need for well-designed prospective investigations to reach definitive conclusions regarding the clinical efficacy and safety of this approach, in order to inform the standardization of protocols and strengthen evidence-based clinical decision-making within the context of supportive care in oncology.

## Data Availability

In line with Open Science principles, this study is a scoping review and did not generate primary data involving human participants or biological samples. All data analyzed were derived from previously published studies and are available in the public domain. The review was conducted using a rigorous methodology based on established scoping review frameworks and was prospectively registered in the Open Science Framework.
